# Current Perspective on the Natural Compounds and Drug Delivery Techniques in Glioblastoma Multiforme

**DOI:** 10.3390/cancers13112765

**Published:** 2021-06-02

**Authors:** Tapan Behl, Aditi Sharma, Lalit Sharma, Aayush Sehgal, Sukhbir Singh, Neelam Sharma, Gokhan Zengin, Simona Bungau, Mirela Marioara Toma, Daniela Gitea, Elena Emilia Babes, Claudia Teodora Judea Pusta, Adrian Gheorghe Bumbu

**Affiliations:** 1 Department of Pharmacology, Chitkara College of Pharmacy, Chitkara University, Rajpura 140401, India; aayush18004.ccp@chitkara.edu.in (A.S.); sukhbir.singh@chitkara.edu.in (S.S.); neelam.mdu@gmail.com (N.S.); 2 Department of Pharmacology, School of Pharmaceutical Sciences, Shoolini University, Solan 173229, India; aditisharma31790@gmail.com (A.S.); lalitluckysharma88@gmail.com (L.S.); 3 Department of Biology, Faculty of Science, Selcuk University, Konya 42130, Turkey; gokhanzengin@selkuc.edu.tr; 4 Department of Pharmacy, Faculty of Medicine and Pharmacy, University of Oradea, 410028 Oradea, Romania; mmtoma@uoradea.ro (M.M.T.); dgitea@uoradea.ro (D.G.); 5 Doctoral School of Biological and Biomedical Sciences, University of Oradea, 410087 Oradea, Romania; 6 Department of Medical Disciplines, Faculty of Medicine and Pharmacy, University of Oradea, 410073 Oradea, Romania; eebabes@uoradea.ro; 7 Department of Morphological Disciplines, Faculty of Medicine and Pharmacy, University of Oradea, 410073 Oradea, Romania; cjudeapusta@uoradea.ro; 8 Department of Surgical Disciplines, Faculty of Medicine and Pharmacy, University of Oradea, 410073 Oradea, Romania; abumbu@uoradea.ro

**Keywords:** glioblastoma multiforme, flavonoids, therapeutic potential, oxidative stress, natural compounds, drug delivery techniques

## Abstract

**Simple Summary:**

Glioblastoma multiforme (GBM) is one of the belligerent neoplasia that metastasize to other brain regions and invade nearby healthy tissues. However, the treatments available are associated with some limitations, such as high variations in solid tumors and deregulation of multiple cellular pathways. The heterogeneity of the GBM tumor and its aggressive infiltration into the nearby tissues makes it difficult to treat. Hence, the development of multimodality therapy that can be more effective, novel, with fewer side effects, improving the prognosis for GBM is highly desired. This review evaluated the use of natural phytoconstituents as an alternative for the development of a new therapeutic strategy. The key aspects of GBM and the potential of drug delivery techniques were also assessed, for tumor site delivery with limited side-effects. These efforts will help to provide better therapeutic options to combat GBM in future.

**Abstract:**

Glioblastoma multiforme (GBM) is one of the debilitating brain tumors, being associated with extremely poor prognosis and short median patient survival. GBM is associated with complex pathogenesis with alterations in various cellular signaling events, that participate in cell proliferation and survival. The impairment in cellular redox pathways leads to tumorigenesis. The current standard pharmacological regimen available for glioblastomas, such as radiotherapy and surgical resection following treatment with chemotherapeutic drug temozolomide, remains fatal, due to drug resistance, metastasis and tumor recurrence. Thus, the demand for an effective therapeutic strategy for GBM remains elusive. Hopefully, novel products from natural compounds are suggested as possible solutions. They protect glial cells by reducing oxidative stress and neuroinflammation, inhibiting proliferation, inducing apoptosis, inhibiting pro-oncogene events and intensifying the potent anti-tumor therapies. Targeting aberrant cellular pathways in the amelioration of GBM could promote the development of new therapeutic options that improve patient quality of life and extend survival. Consequently, our review emphasizes several natural compounds in GBM treatment. We also assessed the potential of drug delivery techniques such as nanoparticles, Gliadel wafers and drug delivery using cellular carriers which could lead to a novel path for the obliteration of GBM.

## 1. Introduction

The incidence of brain tumor has evidently been increasing during the last few decades [1]. The primary brain tumor is mainly termed as “glioma”; its different types are differentiated based on their cellular origin and comprise astrocytic tumors (glioblastoma multiforme (GBM), astrocytoma and anaplastic astrocytoma), oligodendrogliomas, mixed gliomas and ependymomas [2]. Among various brain tumors, GBM is one of the belligerent neoplasia having the highest mortality rate and current therapeutic modalities are mostly palliative [3]. Moreover, it has a survival time period of less than 1 year 3 months, and a 2-year survival time period (between 26% to 30%) after successive resection, radiation and temozolomide chemotherapy [4]. The characteristics of this type of tumor are diffused brain infiltration, increased cell proliferation, necrosis and resistance to available pharmacological therapies [5]. GBM has two origins: primary GBM (i.e., tumors occurring de novo) representing 90–95% of all GBMs, the remaining 5–10% cases are secondarily originating from low-grade tumors, occurring due to multiple genetic mutations in vascular endothelial growth factor receptor (VEGFR), retinoblastoma protein (RB), phosphatase tensin homolog (PTEN) and several other mutations, causing p53inhibition, amplifying platelet-derived growth factor receptor-α (PDGFRα) and overexpressing cyclin-dependent kinase 4 (CDK4) [6].

In terms of morphology, there is a similarity between primary and secondary GBMs, although variation occurs in genetic patterns and the molecular signaling pathways leading to the progression and development. In primary GBM, there is an elevation of epidermal growth factor receptor (EGFR), mutation or deletion of homozygous CDK4, changes in chromosome 10 associated PTEN, etc. [7]. Elevated reactive oxygen species (ROS) in tumor cells cause mitochondrial dysfunction, oncogene activity, peroxisome activity, glucose metabolism and decreased antioxidant system [8]. The somatic and hereditary mutations in oncogenes and tumor suppressor genes result in tumor formation. The chromosomal damage leads to an abnormal cell population. However, the biomolecular pathways initiating tumor growth are still under investigation. The oxidative stress associated with carcinogenic activity depends upon the ROS capacity in causing genotoxicity and obstruction of important cellular events. [9].

Cellular proteins, pyrimidines, purines bind with hydroxyl radicals causing chromosomal alterations and modifications in the expressions of genes. Cancer cell immortalization and telomere maintenance require human telomerase reverse transcriptase (hTERT), (catalytic subunit of the telomerase). In GBM, TERT promoter mutations with enhanced telomerase expression are remarkably very high. Inhibition of hTERT induces apoptosis of glioma cells and abrogates pentose phosphate pathway (PPP). The PPP is the main source of NADPH needed for fatty acid synthesis, as well as for RNA synthesis and de novo lipogenesis cofactors. In gliomas, a key metabolic enzyme, Fatty acid synthase (FASN), that plays an important role in de novo lipogenesis, is over-expressed. In GBM, enhancer of zeste homolog 2 (EZH2), the methyltransferase polycomb group protein is highly expressed. TERT-EZH2 network regulates DNA damage responses and lipid metabolism in glioblastoma. [10].

ROS accumulation in cancer cells causes damage to DNA directly or due to cellular mutation and phenotype enhancement. Furthermore, mutations occurring due to impaired repair processes are seen in tumor suppressor genes such as Ras and P53. P53 plays a crucial role in genomic integrity surveillance and apoptosis regulation [11].

GBM patients have a poor prognosis despite various advancements in diagnosis and treatments. This may result due to resistance in standard chemotherapeutic drugs and ionizing radiation, which may cause cancer stem cells (CSCs) to form a massive tumor mass [12]. The resistance in CSCs is linked with their slow-cycling phenotype, changed profile of cell surface markers, efflux transporters expression, antiapoptotic proteins, DNA damage response and repair pathways, as well as elevated free radical scavengers [13]. Furthermore, the treatments available are associated with adverse reactions, thereby decreasing quality of life [14]. A pharmacological regimen with high efficacy and reduced toxicity is urgently needed.

Natural compounds are found ubiquitous in plants and vegetables and have numerous pharmacological activities, such as antineoplastic, antioxidant, antiviral and anti-inflammatory [15,16,17]. In current years, natural compounds have raised scientists interest, and are scrutinized for pharmaceutical interventions against cancer development [18]. They have a beneficial role in the molecular signaling events of cancer such as cell cycle arrest, DNA topoisomerase I/II suppression, proteasome inhibition, regulation of survival/proliferation events, topoisomerase downregulation, suppression of fatty acid synthesis and p53 accumulation [19,20]. They can cross the endothelial cell tight junctions of BBB by transcellular diffusion and paracellular diffusion. Natural compounds are P-glycoprotein (P-GP) substrates, thus having bidirectional carrier-mediated approach, P-GP is an efflux transporter in the brain that transfers substrate from interstitial fluid (ISF) to the endothelium region [21,22].

This review was possible accessing recognized medical databases and judiciously selecting the most informative, recent and relevant papers on the topic addressed. We consider that an extremely useful, focused and accessible database was obtained, in order to meet the interest of those seeking information on this topic.

The main purpose of this research is to discuss the role of various natural compounds in GBM treatment, compounds that work by increasing the efficacy of chemotherapeutic drugs on the grounds of metastasis, angiogenesis, apoptosis, ER stress, ROS and enhanced pharmacological activity. We also assessed the potential of drug delivery techniques (such as nanoparticles, gliadel wafers and drug delivery using cellular carriers), which could lead us to a novel path for the obliteration of GBM.

## 2. Underlying Mechanisms for GBM Progress

There are various genetic and molecular changes in GBM that cause alterations in various signaling events, resulting in brain tumor progression. It is relevant to mention that the most crucial signaling events involved in GBM are the tyrosine kinase receptor (TKR) family [23]. Ligand-stimulated receptors initiate downstream signal transduction events, such as Ras/Raf/mitogen-activated protein kinase (MAPK), the protein kinase C pathway, the PI3K/AKT signaling pathway and STAT signaling pathway [24]. These signaling events regulate cell survival, proliferation and angiogenesis.

The present research was focused on TKR, PDGFR, EGFR, VEGFR and fibroblast growth factor receptor (FGFR), the hepatocyte growth factor receptor (HGFR) and insulin-like growth factor 1 receptor (IGF-1R) [25]. PDGF plays a crucial part in tumoral gliogenesis. It activates intracellular pathways through G-protein receptors and secondary mediators that link up at various sites. PDGF and EGFR increased expression in GBM shows that TKR cascades are the crucial targets [26]. The four ligands (PDGF-A-D) found in the PDGF family function through the two-cell surface TKR PDGFα and β receptor [27]. PDGF receptor activation is associated with various intracellular pathways among which the Ras-MAPK, PLCγ, PI3K signaling events are important. In primary GBM tissues and glioma cells, the co-expression of PDGF ligands and receptors is commonly observed. This suggests that autocrine as well as paracrine loops together contribute to tumor growth and progression.

In tumor cells, PDGFRA and PDGFA are commonly found and PDGFB and PDGFRB are expressed in glioma-linked endothelial cells. In animal model studies, PDGFA activates the PDGFRA-positive neural stem cell proliferation in the subventricular zone, which differentiates and develops lesions similar to glioma [28]. In wild-type mice, glioma-like lesions resembling human glioblastoma, with massive necrotic areas were seen when recombinant Moloney murine leukemia virus encoding PDGFB was administered in combination with a replication-competent helper virus [29]. In glioblastoma tumors, the amplification, mutation and rearrangement of the PDGFRα gene are seen [30]. This suggests that PDGFα receptor signaling over activity and genetic aberrations are the main events in glioblastoma development. This view is supported by glioblastoma transcriptome analysis which proved an abnormality in the PDGF/PDGFR pathway [31].

EGFR belongs to tyrosine kinases receptor family and its further subtypes include ErbB1, ErbB2 ErbB3 and ErbB4. In 45%–57% of GBM cases, mutations were detected in EGFR, indicating a vital role in the GBM pathogenesis and resistance to therapeutic regimen [32]. In GBM another truncated mutant EGFR variant III is commonly stimulated independent of a ligand, causing cell survival and proliferation. Despite its growth intensifying properties, its expression is associated with an elevated survival rate in GBM patients. EGFRvIII is a neoantigen resulting in immune response elicitation [33].

In glioma cells, the presence of these receptors dramatically increases tumor formation in vivo via enhanced cellular proliferation and decreased apoptosis. In angiogenesis, endothelial cells from the already existing vascular network, sprout and proliferate forming new blood vessels, whereas in vasculogenesis there is a formation of de novo blood vessels by circulating progenitor endothelial cells that recruit into the tumor [34]. These two mechanisms are orchestrated via VEGF and its associated receptor 2 (VEGFR-2), IL-8 and -6, FGF, as well as angiopoietins [35]. In normal brains, expression of endothelial VEGFR-2 was found to be low or undetectable; however, the levels of VEGFR-2 vessels and the expression of endothelial VEGFR-2 elevate with tumor grade, more in GBM [36].

Additionally, in another study VEGFR-2 expression mainly depends on the inflammatory state of the tumor: the endothelial VEGFR-2 expression increases as the inflammation rises. In the inflammatory infiltrate, the presence of macrophages forms numerous angiogenic mediators and cytokines, forming blood channels via proteolytic pathways thereby stimulating angiogenesis [37].

### 2.1. The PI3K/AKT/mTOR Signaling

This signaling pathway is stimulated by the transmembrane tyrosine kinase growth factor receptor family, G-protein-coupled receptors and transmembrane integrins. In the GBM pathogenesis elevated PI3K-mediated cell signaling was implicated. Based on subsequence homology and substrate specificity, PI3K is broadly divided into three types, among all, class I plays a vital role in tumorigenesis. Class I has a subunit p85 as well as catalytic subunit classified as p110 (α, β, γ) and fourth isoform (p110δ) is combined with the p101 regulatory subunit and comes under category PI3Ks IB [38]. Upon receptor activation through phosphorylated tyrosine will interact with p85 resulting in a change of subsequent conformation and releasing catalytic subunit p110. The catalytic subunit p110 forms phosphatidylinositol 3,4,5-triphosphate (PIP3) from precursor phosphatidylinositol bisphosphate. PIP3 further leads to the recruitment of downstream effector molecules like phosphoinositide-dependent kinase 1 (PDK1) and Akt [39]. Activated Akt leads to the stimulation of the downstream mammalian target of rapamycin (mTOR) regulated response to protein and ribosome biogenesis. In PI3K signaling cascade, mTOR acts like downstream effector molecule as well as an upstream modulator [40]. It resides in mTORC1 and mTORC2 [41]. The mTORC1 consists of mTOR, mLST8, PRAS40 and Raptor.

**Figure 1:**
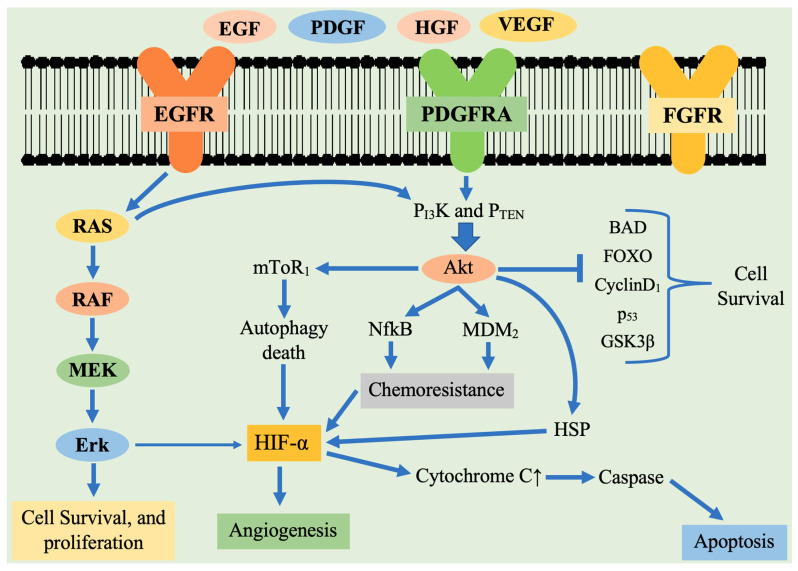
Schematic representation of tyrosine kinase receptor activation and the major downstream signaling pathways involved in the pathogenesis of glioblastoma multiforme. Receptor tyrosine kinase are activated by ligand binding causing receptor dimerization and auto phosphorylation of the tyrosine kinase domain. This results in activation of two main downstream signaling pathways: Ras/ERK and PI3K/AKT. These receptors activate downstream signaling cascade that participates in cell survival, proliferation, angiogenesis. Legend: EGF—endothelial growth factor; EGFR—epidermal growth factor receptor; PDGF—platelet derived growth factor; PDGFR—platelet derived growth factor receptor; VEGF—vascular endothelial growth factor; VEGFR—vascular endothelial growth factor receptor; mTOR 1—mammalian target of rapamycin 1; HIF1α—hypoxia-inducible factor 1α; HSF—Heat shock protein; PTEN—phosphatase tensin homolog; MMP-2—matrix metallopeptidase 2; PI3K—Phosphoinositide 3-kinases; NFκB—nuclear factor κB; MAPK: Mitogen—activated protein kinase; MEK—MAPK extracellular signaling-regulated kinase; FOXO—Forkhead box O; FGFR—fibroblast growth factor receptor; BAX—BCL2-associated X protein; MDM2—murine double minute 2 ERK Extracellular regulated kinase; p53—tumor protein.

Cell proliferation and growth are regulated through mTORC1 mediated S6K1 and S6 activation. It also inhibits eukaryotic initiation factor 4E (eIF4E), binding protein 1 (eIFBP1), consequently leading to the formation of eukaryotic initiation factor 4F [42]. The composition of mTORC2 includes mTOR, Sin1, Rictor and mLST8. It has been suggested that mTORC2 initiates PKC activity [43]. The mTORC2 also has a role in cytoskeletal organization and cell survival. The mTOR modulates hypoxia-inducible factor 1α (HIF1α), inducing downstream signaling of VEGF and upregulates angiogenesis [44]. The Akt activation blocks tuberous sclerosis complex (TSC) 1/2, thus activating mTORC1-regulated events which involve phosphorylation of ribosomal protein S6 kinase (pS6k), eIF4E and eIFBP1, involved in protein translation, ribosomal biogenesis, mediating cell growth [45]. The mTORC2 is involved in Akt phosphorylation at Ser-473, playing a vital role in cell survival, proliferation and cytoskeletal organization [46]. Akt activation also leads to phosphorylation of the FOXO subfamily, thereby inhibiting pro-apoptotic proteins (BAD and GSK3) transcription [47]. It also phosphorylates and degrades the inhibitor of κB (IκB), thereby enhancing the activity of NF-κβ and activation of pro-survival genes. Activated Akt also regulates MDM2 activity which blocks P53 (cell-cycle arrest activator). In PI3K signaling cascade, another crucial molecule is PTEN (Figure 1).

Previous reports also showed that PTEN has an important role in causing G1 phase cell cycle arrest. The EGFR or PTEN alteration induces continuous stimulation of the PI3K/Akt/mTOR pathway and increases activated AKT levels in glioma cells, thereby leading to tumorigenesis and resistance to cancer therapy [48].

### 2.2. The Ras Pathway

Ras is a member of the G-protein family, thereby its stimulation or deactivation is under the control of guanosine triphosphate (GTP) or with guanosine diphosphate (GDP), respectively [49]. Cell surface receptors activate this signaling pathway and regulate the functioning of numerous cellular events participating in angiogenesis, cell migration, proliferation and survival. Activated RAS stimulates RAF kinase, which further causes the activation of downstream events which include MAPK subsequently PI3K pathway and Ral-guanine nucleotide exchange factors [50]. This signaling pathway is stimulated in some tumors by cytokine receptor mutation which includes Flt-3, Fms, Kit or mutated receptors overexpression [51]. Ras/MAP/ERK pathway activates HIF-1α, which leads to tumorigenesis [52]. In GBM, the RAS pathway overexpression was detected. In addition, EGFR, PDGFR or TKRs that regulate RAS are also overexpressed, implying a correlation between PDGFR expression and RTKs in GBM pathogenesis [53]. Thus, the RAS/MAPK signaling pathway can be targeted in designing a therapeutic regimen for GBM.

### 2.3. The p53 Pathway

In cancer, p53 represents one of the dysregulated genes in GBM, hematopoietic cancers and ovary carcinoma [54]. According to the Cancer Genome Atlas, the abnormality in the ARF-MDM2-p53 signaling pathway is seen in 84% of GBM cases and 94.1% in GBM cell lines [55]. P53 shows tumor suppressor activity mainly by modulating the cell cycle arrest, cellular senescence and apoptosis [56]. It is primarily stimulated in DNA damage, during activated oncogenes, genotoxicity, hypoxia and abnormal growth signals. These events can be encountered during the process of carcinogenesis [57]. MDM2 (E3 ubiquitin ligase) is a vital modulator of the p53 signaling cascade. It controls p53 by inhibiting transcription and E3 ligase degradation [58]. MDM2 overexpression occurs in about 10% GBMs cases, but only in those tumors having no p53 mutation, suggesting that MDM2 overexpression can be an alternative path for tumors to block p53 mediated growth control but without altering p53 expression [59]. Another vital regulator of p53 is MDM4. Due to its upregulation in malignant gliomas, it has been a major area of interest due to its beneficial pharmacological effects [60]. One previous report indicated that MDM4 overexpression results in GBM through a p53-independent growth control signaling cascade same as seen in the MDM2 signaling cascade [59,60,61].

### 2.4. Interleukin-6 (IL-6)/STAT3 Pathway

IL-6 belongs to the cytokine family that regulates immunity and inflammatory responses. Intriguing, the expression of IL-6 was seen in many malignant tumors like breast cancer, lung cancer, GBM and prostate cancer [62]. Furthermore, IL-6 has a crucial role in GBM [63]. IL-6 binds via interleukin receptor alpha subunit IL-6Rα, that formed IL-6/IL-6Rα complex binds with IL-6 co-receptor gp130, resulting a hexameric complex. The IL-6 mediated signaling is of two types: classical and trans-signaling. Signaling through the classical pathway is the main membrane-bound IL-6R signaling that is highly seen in few tissues like hepatic cells, leukocytes and epithelial cells [64]. On the contrary, IL-6 trans-signaling occurs in many cell types, as a ubiquitous expression of gp130. This results in soluble IL-6Rα (SIL-6Rα), trans-signaling that does not have a transmembrane domain, via binding with gp130. Both signaling events induce JAK proteins activation, further this intracellular signaling forms the platform for phosphorylation of STAT transcription factors, particularly STAT3 [65]. The STAT protein family plays a key role in the nuclear signal transmission for the transcription of several genes. Owing to the oncogenic property of STAT3 it has a vital role in cancer. STAT3 increases the expression of genes that can facilitate angiogenesis, tumor survival, cell cycle progression and resistance to cell death. The genes that STAT3 targets include VEGF, Bcl-2, Bcl-xL, cyclin D1, c-myc and human telomerase reverse transcriptase [66]. Additionally, the IL-6 pathway correlates with the upregulation of fascin-1, which participates in cell invasion by forming actin-based protrusions called invadopodia and further in disruption of the extracellular matrix leading to migration and invasion [67]. This process causes the release of mediators which include Matrix metallopeptidase 9 (MMP-9), Matrix metallopeptidase 2 (MMP-2), Tumor necrosis factor-beta (TNF-β), Tumor necrosis factor-alpha (TNF-α) and VEGF [68]. The STAT3 transcription factor increases VEGF-2, VEGFR-2, expression promoting invasion via IL-6 JAK-STAT3 signaling pathway [69]. In ganglioglioma there is a downregulation of miR-217 which is concomitant with increased levels of casein kinase (CK-2α) and Erk levels. CK-2α affects miR-217/ERK/mGluR1 interplay in long term tumors associated with epilepsy [70].

### 2.5. Role of Glutamate Receptors in GBM

In the CNS, neurons show the presence of ligand-gated ion channels, which are stimulated by alterations in membrane voltage, or by interaction with intracellular ligands like Ca^2+^ and cyclic nucleotides, or by extracellular neurotransmitters or both. GBM cells express receptors and ion channels which are of great interest for their vital roles in signaling cascades that promote lethal glioblastoma tumor development [71]. Among diverse ion channels, the transcript levels were highest for glutamate receptor genes, and low levels of nicotinic acetylcholine and GABA_A_ receptors. In the brain and spinal cord regions, Glutamate receptors are the main excitatory neurotransmitters released in response to an elevated intracellular Ca^2+^ level via voltage-gated Ca^2+^ channels and leading to an excitatory response in membrane potential [72]. Glutamate receptors have been subdivided into the N-methyl-D-aspartate (NMDA) and AMPA alpha-amino-3-hydroxy-5-methyl-4-isoxazole propionic acid). In neurons, the AMPA receptors, Ca^2+^ permeability is negligible while in the malignant tumor, there is a decreased efficiency [73]. In pediatric GBM cases, 50% to 70% of the GluA2 RNA, that augment the Ca^2+^ pathways leading to proliferation and migration of glioblastoma [74].

### 2.6. Role of Voltage-Gated Ion Channels in GBM

Voltage-gated Ca^2+^ channels (Cav) are implicated in various functions, such as controlling the Ca^2+^ entry, release of hormones and neurotransmitters. Ca_V_ channels have numerous subunits that are encoded by different genes; among which the ion pore is formed from α-1 subunit forms and is linked with α-2 subunits, β, γ and δ forms that control functional activities. In glioblastoma, the Ca^2+^ channel-associated genes show transcript levels [71]. At the RNA level, in the glioblastoma patients, the expression of voltage-gated Na^+^ (Na_V_) 1, 2, and 3 α subunits were seen through transcriptomic data. The main part of Na_V_ channels is action potential generation, that travels over distances, and through the Na^+^ gradient, controls the regenerative loop. The action potential is further linked to the voltage-gated arrival of Ca^2+^ to generate neurotransmitter release, hormone secretion and muscle contraction. In GBM cells, measurable action potentials were absent [75].

There is a possibility that Na_V_ protein might be non-functional or not assembled, its plasma membrane distribution might not have spatial organization, might cause undetectable depolarization reaction. Blockers of the Na_V_ channel were found to decrease migration and invasiveness in the U87 GBM cell line. This is highly independent of the inhibition of Na^+^ channel, as tetrodotoxin application was not able to replicate it, thereby it was concluded to be a RNA demethylase side effect [76]. In glioblastoma, Na_V_ channels might cause excitatory responses, but still, their logical role remains to be tested in the future. In various types of cells, voltage-gated K^+^ channels play important role in maintaining negative resting membrane potential, and also during the repolarization phase, they restore the resting voltage responses in neurons, sensory cells and muscles [71].

### 2.7. Oxidative Stress in Glioblastoma Multiforme

ROS are the compounds of aerobic metabolism, few examples include hydrogen peroxide (H_2_O_2_) hydroxyl radical (OH), and superoxide anion (O_2_^−^). However, imbalance in endogenous ROS levels and antioxidant molecules causes membrane lipid, protein and DNA damage leading to cell death [77]. Thus, the antioxidant balance influences cell fate [78]. Cancer cells show a high glycolysis rate with decreased respiration rate [79]. The increased need for ATP leads to oxidative stress which eventually induces cell death. The cytochrome c release and its coupling with the pentose phosphate initiate cytochrome c regulated apoptosis [80].

During apoptosis, the activation of caspase is also initiated by cytochrome c in mitochondria. These marked alterations in metabolism are related to raising oxidative stress which is due to the upsurge production of mitochondrial superoxide radical [81]. In the brain, oxidative stress raises glutamate level in the hippocampus region that affects neurotransmission. In neurons, the activated glutamate receptors consequently result in acidification because of Ca^2+^ entry that leads to the death of neurons centrally [82]. The antioxidant enzyme was evaluated in multiple brain tumors, however, studies mainly emphasized reduced antioxidant enzyme levels and vitamins in different malignancies [83].

Various factors affect the cell response in ROS-associated damage, it highly depends on the duration and intensity of the stimulus [63]. By binding with proteins, ROS affects different mechanistic pathways that have an important role in cell proliferation and apoptosis. The downstream pathways generally involve oxidation of cysteine and/or tyrosine residues, altering the role of various proteins. These alterations are reversible and promote cellular responses [84]. Through a process of phosphorylation, ROS can alter the signaling protein structure and thereby could indirectly impede the signal process, which includes immune signaling, aging, metabolism, hypoxic stress and apoptosis [63]. Specifically, ROS can accelerate the EGF and PDGF-mediated signaling events [77]. ROS inhibits phosphatases, whereas kinases may be activated or inhibited [85]. ROS stimulates nonreceptor protein kinases like G proteins, Ras, TKR and the components of JNK and p38 kinase cascade that promote apoptosis [86]. Particularly, through disulfide bond formation among catalytic cysteine, H_2_O_2_ blocks phosphatase, and PTEN [87]. It also oxidizes thioredoxin, a redox protein, thereby inhibiting its suppressing effect on the p38MAPK pathway [88].

Most ROS-sensitive signaling pathways affect various transcription factors by transducing cytoplasmic signals towards the nucleus, thus controlling the wide array of gene expression. The nuclear factor erythroid 2 (NF-E2-) related factor 2 (NRF2) regulates the antioxidant responses in cancer cells. It is one of the important events through which tumors counteract ROS. NRF2 regulates the enzymes, having the main role on the components of endogenous antioxidants [89].

In unstressed conditions, NRF2 is associated with KEAP1 being concurrently degraded upon ubiquitination. As the cell undergoes exposure to radiation or chemical toxins, there is a dissociation NRF2 KEAP1complex that aggregates in the nucleus and stimulates various antioxidant genes, that protect cells from DNA damage [90]. Moreover, NRF2 controls protein expression that leads to GSH and superoxide dismutase (SOD) homeostasis. SOD is vital for the superoxide anion radical dismutation into H_2_O_2_ and oxygen. SGK1, an upstream regulator to NRF2, promotes antioxidant stimulation [91]. In cancer cells, glucose metabolism and ROS homeostasis are affected by Nrf2-regulated PPP. In glioma, TERT promoter mutations was considered as potential biomarker and mitochondrial localization of hTERT induced by oxidative stress is known to trigger apoptosis. Nrf2 regulates PPP, thereby promoting tumorigenesis. GBM patient tumors with TERT mutations lead to the activation of telomerase also revealing the elevated levels of TKT and Nrf2 and diminished glycogen deposition. In GBM patients, Nrf2-TERT regulatory loop promotes progression of glioma by affecting cell survival, metabolism and redox homeostasis [92].

Genomic analyses suggest phenotypic alterations in cholesterol homeostasis pathways in glioblastoma. It includes increased gene copy numbers and cholesterol synthesis gene expression, enhanced cholesterol imported by LDL receptors and reduced transport of cholesterol. They all together promote increased content of cellular cholesterol that leads to cancer cell proliferation [93].

In glioma stem cells PI3K/AKT signaling pathway upregulation leads to tumor formation [94]. NRF2 suppression reduces GBM proliferation or causes sensitization to chemotherapeutic treatment [95]. The Nrf2 knockdown decreases the proliferation of glioma cells via ATP depletion, 5′ AMP-stimulated PK activation and subsequently blocks the mTOR pathway [96]. The mTOR, a vital regulator of the PI3K event, is a vital hallmark in GBM, although pharmacological regimens to target the Nrf2/mTOR cascade are not yet found. Collectively, a thorough knowledge of the Nrf2 suggests that survival of tumor cells can be possible through escalating their antioxidant capacity, as well as molecular adaptation providing a higher capacity for inactivating drugs. Therefore, suppressing the Nrf2 pathway and overcoming the chemoresistance appears to have potential [97]. Another vital transcription factor is p53, that controls antioxidant gene expression. Indeed, p53 leads to both antioxidant and oxidant gene expression, thereby its role in regulating ROS is controversial. Moderately increased levels of ROS block p53, while elevated levels induce its expression. The p53 targets are sestrins that promote the peroxiredoxins activity, upregulating the cellular levels of antioxidants [98,99]. In particular, p53 and FOXO regulate genes that are not linked to Nrf2, although these factors promote HMOX1 expression, thereby storage and release of Fe(II), which plays an important part in tumorigenesis [100].

In tumorigenesis, abnormal cellular metabolism is a common feature as revealed by numerous studies [101]. In GBM abnormal energy metabolism and metabolic reprogramming are considered as a hallmark of tumors [102]. The source of energy for glioblastoma cells is via glycolysis [103]. The glycolysis upregulation due to increased consumption of glucose was first evidenced by Warburg [104], and seems to be the main feature of primary and GSCs [105]. In various tumors, TGF-β and human chorionic gonadotropin β (hCG-β) are known as regulators of oxidative stress and survival responses. In GBM tumors, TGF-β treatment increased hCG-β content. Increased hCG-β regulates redox homeostasis in glioma cells treated with TGF-β. The hCG-β knockdown mediated by siRNA (i) increases ROS (ii) reduces expression of thioredoxin Trx1 and thioredoxin reductase (TrxR) activity (iii) abrogates TP53-induced glycolysis expression and apoptosis regulator (TIGAR). The hCG-β silencing abrogated Smad2/3 levels, demonstrating the existence of TGF-β hCG-β cross-talk in GBM cells [106].

To satisfy the tumor cell energy, an ample amount of glucose is needed for their survival and growth. During hypoxic stress, pyruvate dehydrogenase kinase 1 is administered to reduce the ROS production in GBM cells [107]. The miR-210 transcription is caused by HIF, this microRNA elevates the caspase-3/-7 activity, apoptotic rate and reduces invasive capacity, lactate production, ROS, in hypoxic GSCs [108]. On the contrary, higher HIF-1 levels may cause glycolytic enzymes that promote lactate production [109]. This biochemical conversion from OXPHOS to anaerobic glycolysis is the leading hallmark of GBM. HIF-1α expression is controlled by glycolytic enzyme pyruvate kinase M2 by promoting its interaction with hormone response element. The HIF-1 cascade reduces cytochrome c oxidase assembly protein and iron-sulfur cluster assembly proteins, two vital components of the tricarboxylic acid (TCA) cycle and the mitochondrial electron transport chain (ETC). HIF-1α activation can upregulate the Bcl-2 expression to hinder cell apoptosis.

The oncogenic events (like MAPK/ERK, PI3K/AKT and STAT3 pathways) also lead to transcriptional HIF-1α expression, to raise the glucose utilization in tumors even during sufficient oxygen. Thereby, HIF-1 is important during hypoxic conditions to decrease mitochondrial respiration, electrons leakage in ETC and also suppressing ROS formation thus ensuring the GBM cells survival [110]. HIF-1α targeting via the HIF-1 pathway could regulate metabolic enzymes involved in cancer. This may prove to be beneficial in the development of selective and effective GBM therapy.

### 2.8. Reactive Oxygen Species and Apoptosis

ROS kills transformed cells through programmed cell death (PCD), which is achieved within less than 60 min through caspases. This is achieved by an intrinsic or extrinsic cascade, caspase-PCD ends with apoptotic body formation which is eradicated by adjoining phagocytes [111]. The extrinsic signaling pathway is controlled by TNFα. Fas ligand binds to its cognate receptors triggering recruitment of adaptor proteins and pro-caspases. This promotes the formation of death-inducing signaling complex (DISC) and caspases [112]. Evidence showed that anticancer drugs targeting ROS highly depend on the intrinsic pathway. This pathway includes mitochondrial PTPs and the production of pro-apoptotic factors like cytochrome c; it also increases the permeability which leads to the pro-caspase 9 complexes with apoptotic protease by activating factor 1 forming apoptosome. In turn, it activates effector caspases [113]. Indeed, ROS produces three important components for the mPTPs opening, the voltage-dependent anion-selective channel, cyclophilin D and adenine nucleotide translocase through the oxidation of particular cysteine [114]. ROS also activates apoptosis by upregulating the Bcl-2 expression and by reducing Bax and Bad levels [115].

ROS-induced autophagy is the vital pharmacological approach for cancer cell apoptosis [116]. Hydrogen peroxide (H_2_O_2_) inhibits autophagy-associated gene-4 and upregulates LC3-related autophagosomes [117]. Autophagy is a cell survival pathway being also considered a tumor suppressor event that leads to the death of mutated cells [118]. In glioma cells, H_2_O_2_ causes autophagic apoptosis following the polycyclic ammonium ion sanguinarine, a treatment that escalates mitochondrial electron leakage and promotes NOXs expression [119]. Administration of rapamycin, in combination with HSP90 inhibitors, leads to mitochondrial damage, autophagy, oxidative stress and decreases tumor growth [120].

ROS can also induce necrosis, recognized as an uncontrolled and passive type of cell death. It is also considered as necroptosis or type III programmed cell death [121]. The ROS generated from ceramide production or following elevated energy metabolism caused by various protein kinases or mitochondrial ETC enhances necroptosis [122]. Furthermore, the ROS-associated molecular pathway of tumor inhibition via p53 causes cell death, known as ferroptosis.

**Figure 2:**
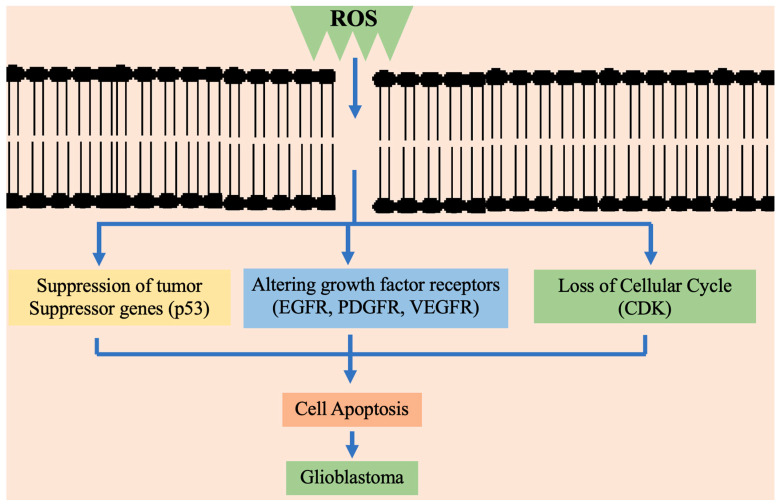
Schematic representation of ROS affecting different mechanistic pathways in cell proliferation and apoptosis in glioblastoma multiforme. EGFR—epidermal growth factor receptor; PDGFR—platelet-derived growth factor A receptor; VEGFR—vascular endothelial growth factor; CDK—cyclin-dependent kinase.

Figure 2 briefly presents the way high ROS concentration consequently blocks the cysteine uptake. It is typically regulated by the suppression of cysteine/glutamate antiporter [123].

## 3. Natural Compounds in GBM

GBM tumors metastasize to other brain regions and invade nearby healthy tissues. Their dissemination to the outside brain regions is halted by the blood-brain barrier (BBB) [124]. However, a fatal outcome appears within the brain regions promoting brain herniation that subsequently disables major brain areas [125]. Conventional chemotherapy and radiotherapy damage the DNA of cancer cells exerting cytotoxic effects. However, these treatments are associated with some limitations when used as single therapy due to the high variations in solid tumors and deregulation of multiple cellular pathways. The heterogeneity of the GBM tumor and its aggressive infiltration into the nearby tissues makes it difficult to treat.

**Figure 3:**
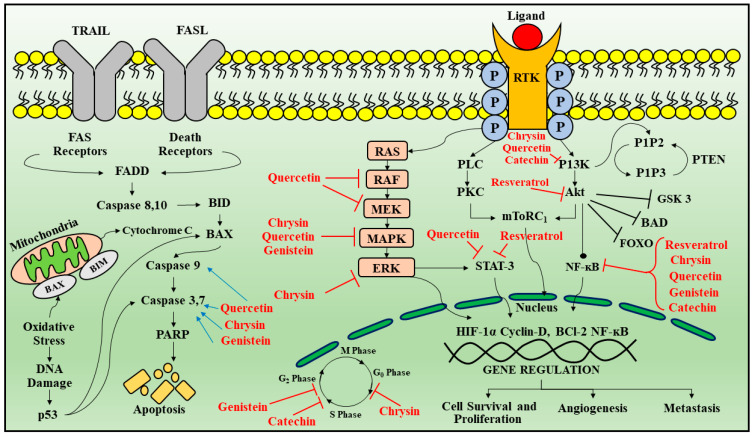
Signal transduction pathways targeted by phytoconstituents in glioblastoma. Apoptotic pathways are regulated by death receptor ligands (TRAIL and FAS). Once activated leads to the activation of FADD which further leads to the activation of caspase 8, 10, that targets BID protein inducing BAX oligomerization and release of cytochrome C from mitochondria along with activation of caspase 3 and 7 causing apoptosis. Another protein that regulates apoptosis is p53 which activates BAX. Receptor tyrosine kinase is activated by ligand binding causing receptor dimerization and auto phosphorylation of the tyrosine kinase domain. This results in activation of two main downstream signaling pathways: Ras/MAPK/ERK and PI3K/AKT. As these receptors activate downstream signaling cascade that participates in cell survival proliferation, angiogenesis and metastasis. Red arrows indicate inhibition and blue arrows indicate activation by phytoconstituents. Legend: TRAIL—Tumor necrosis factor-related apoptosis-inducing ligand; FADD—Fas-associated death domain; BID—B cell lymphoma-2-interacting domain; PARP—Poly (ADP-ribose) polymerase; EGF—endothelial growth factor; PDGF—platelet derived growth factor; HDF—hepatocyte growth factor; VEGF—vascular endothelial growth factor; EGFR—epidermal growth factor receptor; PDGFRα—platelet derived growth factor A receptor-α; FGFR—fibroblast growth factor receptor; PLC—Phospholipase C; PI3K—Phosphoinositide 3-kinases; mTOR 1—mammalian target of rapamycin 1; MAPK—mitogen-activated protein kinase; ERK—Extracellular-signal-regulated kinase; HIF1α—hypoxia-inducible factor 1α; MMP-2—matrix metallopeptidase 2; PTEN—phosphatase tensin homolog; PI3K—phospho inositol 3 Kinase; NFκB—nuclear factor κB.

Hence, there is a need for the development of multimodality therapy that can be more effective, novel, induce fewer side effects, target and improve the prognosis for GBM [126]. Phytochemicals including isoflavones, terpenes, flavonoids and carotenoids, represent a good alternative for the development of a new therapeutic strategy because they are easily obtained and considered as safe, having no toxicity on long-duration. The various sites of regulation by phytoconstituents are summarized in Table 1 and Figure 3 [127,128].

### 3.1. Quercetin

Quercetin (3,3′,4′,5,7-pentahydroxy-flavone) belongs to the flavonols, most common flavonoids found in dietary supplements, apples, grapes, cherries, onions and spinach [125,126]. Quercetin can bind to the transition metal ions and scavenge free radicals and, thereby plays the role of powerful antioxidant [188]. Quercetin has antioxidant property via scavenging reactive nitrogen species (RNS), ROS, antitumor and anti-inflammatory activities, by regulating several signaling events and gene expression in multiple cells, animal models and humans [189]. The anticancer effect of quercetin is through regulating PI3K/Akt/mTOR, heat shock protein (HSP) expression, IL-6/signal transducer and STAT signaling pathways, regulation of apoptosis proteins, altering intracellular pH, as well as VEGF and fibronectin regulation [190].

Quercetin downregulates the activity of PI3K. In U87 and U251 GBM cells, quercetin reduced the Akt protein. The efficacy of TMZ treatment was increased by quercetin when compared to chemoradiotherapy via downregulation of the PI3K/Akt activity [191]. In support of previous studies, a docking of quercetin was shown into PI3K site with—35.59 kJ/mol ChemScore function when compared with its blocker (−31.35 kJ/mol), validating the PI3K blocking by quercetin. In human GBM T98G cells, quercetin causes apoptosis by stimulating the mitochondrial death signaling events. Apoptotic pathways are regulated by death receptor ligands. Once activated leads to the activation of FADD which further leads to the activation of caspase 8, 10, that targets BID protein inducing BAX oligomerization and release of cytochrome C from mitocondria along with activation of caspase 3 and 7, causing apoptosis. Another protein that regulates apoptosis is p53 which activates BAX. Quercetin exposure leads to caspases 3 and 9 activations, causing a release of cytochrome c from the mitochondria also reducing the mitochondrial membrane potential [129].

Quercetin blocks HSF which plays a role in the mitochondrial apoptotic events [130,131]. Quercetin sensitizes GBM cells by downregulating the apoptosis inhibitor protein survivin [132]. Quercetin and caspase blockers upregulate the caspase, thereby, cell viability loss is prevented. Quercetin reduces the survivin expression, antiapoptotic proteins, indicating that quercetin acts through the caspase-dependent pathway by downregulating survivin, extracellular-signal-regulated kinase (ERK) and causing GBM cell death [133]. Phospholipase D (PLD) known as a tumorigenesis regulator, indicates that its inhibition could have an advantage in PLD-regulated GBM pathogenesis [134]. Quercetin blocks the PLD1 expression. Moreover, quercetin downregulates the NFκB-associated PLD1 expression by suppressing NF-κB transactivation [135].

During the process of cellular transport, molecular chaperones help unfolded polypeptides under abnormal metabolic conditions by safeguarding them during stressful situations that induce unfolding in them. In a wide array of tumors, the HSPs are overexpressed [136]. Increased HSP expression in cancers usually increased resistance to therapeutic strategies and portended a poor prognosis. HSP higher expression in transformed cells has a key role in apoptosis suppression leading to resistance to treatment and tumor progression [137]. In the cancer cell, HSP high levels are reinforced by HSF1 hyperactivation, which aids in invasion and metastasis. In many cancers cell lines, quercetin has targeted HSF1-dependent HSPs indicating antitumor activity. In the A549 NSCLC cell line, quercetin inhibits HSP27 causing decreased cell viability [135]. Western blot and immunohistochemical studies have displayed elevated levels of HSP27 in meningioma patient tissues [138]. In addition, overexpression of HSP27 can promote carcinogenesis, multidrug resistance, apoptotic cell death inhibition through direct binding with multiple apoptotic proteins [139]. Furthermore, quercetin combined with cisplatin or gemcitabine displayed more potent cytotoxic activity [140]. Unregulated JAK/STAT signaling contributes to proliferation, survival, inflammation, invasion, new blood vessel formation and metastasis, which are implicated in cancer initiation, progression and advancement. Numerous studies have shown that patient glioma samples frequently display constitutive activation of NF-κB and JAK2/STAT3 signaling pathways [192,193]. Cancer-related inflammatory cytokine IL-6 regulates activation of STAT3 and is upregulated in glioblastoma. Quercetin resulted in the reduction JAK and STAT3 activation [141].

In T98G and U87 cell lines, quercetin blocked IL-6 associated phosphorylation of JAK/STAT3, MMP-2 secretion and cyclin D1 expression and thereby reducing the proliferation, migration and cell cycle arrest. Quercetin is included in clinical trials and is considered a potent chemo-sensitizer [141].

Despite numerous studies on quercetin, clarification of the mechanism of action as a single therapy or in combination with standard chemotherapeutic strategies is still needed in the future, particularly in vivo investigations and clinical trials.

### 3.2. Chrysin

Chrysin (5,7-dihydroxyflavone) belongs to the class of flavonoids obtained from *Passiflora incarnate*, *Passiflora caerulea* [142]. Chrysin acts through molecular events and various inflammatory pathways (p38/MAPK TBK1, Wnt/β-catenin and NFkB) and cell signaling events (AKT/AMPK/ERK/PPAR) [143]. In C6 glioma cells, Chrysin is reported for G1 cell cycle arrest via stimulation of p38/MAPK pathway that causes p21Waf1/Cip1 protein aggregation, or through proteasome activity inhibition [144]. Various studies demonstrated that, in GBM cell lines, Chrysin downregulates ErK/Nrf2 pathway thereby suppressing tumor invasion and migration [145]. Chrysin suppresses Nrf2 in anaplastic glioma, further suppressing the expression of NADPH quinine oxidoreductase-1 and heme oxygenase-1 [145]. In addition, chrysin reduces ROS and increases glutathione peroxidase, superoxide dismutase and catalase activity in the mice model [146]. Chrysin and silibinin combination was efficacious in acute promyelocytic leukemia but showed low sensitivity in GBM [147]. Chrysin significantly downregulates Nrf2 expression at both the mRNA and protein levels via reducing PI3K-Akt and ERK pathway, thereby anticancer drug resistance decreases [148]. Apoptosis induced by chrysin is associated with Akt dephosphorylation in the PI3K signaling pathway [149].

In humans, despite its increased therapeutic benefits, chrysin has reduced bioavailability due to its acute metabolism. The metabolizing enzymes have a high affinity for chrysin, showing its limited oral bioavailability [150]. Various studies showed that some novel dosage forms, like nanoparticles micelles and liposomes as carriers are needed to increase its bioavailability [151].

### 3.3. Luteolin

Luteolin (3,4,5,7-tetrahydroxy flavone) is particularly found in carrots, parsley celery, onion leaves, broccoli, chrysanthemum flowers and sweet bell peppers [152]. The antioncogenic potential of luteolin is through its capacity to suppress cell growth, induce apoptosis and reduce iNOS expression. Luteolin could lead to glioma cell apoptosis via ROS/ER stress pathway and mitochondrial dysfunction [153]. Luteolin triggers cell apoptosis through upregulation of miR-7-1-3p [154]. It also downregulates the EGFR mRNA expression to block cell proliferation in glioma cells [155]. Luteolin and silibinin combination, showed inhibitory activity against U87MG and human glioblastoma T98 G cell lines via (1) growth cells inhibition (2) apoptosis induction (3) downregulation of invasion and migration, (4) blocking of PKCα, (5) decreasing iNOS (6) upregulation of miR-7-1-3p [156]. Moreover, these compounds inhibited the angiogenesis events by apoptosis induction, and by suppressing the PKCα, iNOS and XIAP expressions [157]. Luteolin has also been shown to inhibit the IL-1β, p65, NF-κB, c-Jun amino-terminal kinase. It also blocked the p-AKT and activated caspase-3 and glucose-associated protein. These events were triggered by IL-1β, causing enhanced NF-κB nuclear translocation. Subsequently, luteolin downregulates the IL-1 expression [158].

### 3.4. Genistein

Genistein (5,7-dihydroxy-3-(4-hydroxyphenyl) chromen-4-one) particularly found in *Psoralea corylifolia* and *Pueraria lobata* [160]. It is known to be a phytoestrogen having anticancer potential in various types of cancers, like prostate and breast cancers as well as non-hormonal cancers, like colon carcinoma [161]. Genistein inhibits NF-κB via Akt down-regulation, which is the important apoptosis mechanism in human prostate cancer [162]. Moreover, in in vitro studies, genistein treatment decreased Bcl-2 and elevated Bax, caspase-3,9.

In addition, autophagy induction also has a vital role in the chemo-preventive pathway of genistein, against various tumor types [163]. Rapamycin and genistein combination in U87 human GBM cells demonstrate downregulation of Akt phosphorylation and its associated downstream mTOR pathway [164]. Genistein was also reported to exhibit growth arrest in A172 and ONS76 radiosensitive GBM cells, while this growth arrest was absent in radio-resistant cells, which suggests that genistein possesses chemo-preventive effect, not cytotoxic potential, and thereby it could be a potential component for combined chemotherapeutic regimen [78]. A previous study of genistein combined with carmustine in glioma cell lines such as U87 and C6 reported the synergistic growth inhibitory activity [165]. Genistein upregulates the efficacy of chemotherapeutic drugs (carboplatin, tamoxifen) and other flavonoids, like epigallocatechin gallate and quercetin [166]. Genistein causes cytotoxicity in LN308, LN18 and T98G GBM cells via topoisomerase II inhibition and G2/M phase arrest, increasing tumor suppressor genes, like p21 [167]. Genistein also downregulates the transcriptional events of telomerase reverse transcriptase, which have a crucial role in encoding the catalytic site of telomerase in GBM and other neuroblastoma cells. Genistein inhibits the growth of tumor cells invasion, via tyrosine kinase EGFR inhibition and also blocks urokinase plasminogen activator that plays a role in multiple activities of the metastatic cascade [168].

### 3.5. Catechins

Catechins belong to flavan-3-ols class and are the eponymous flavonoid catechin derivatives [170]. The important sources are green and black tea, red wine, custard apples, broad beans, peaches, apples, plums and cherries [171]. Catechins have decreased bioavailability due to slow absorption and increased first-pass metabolism and high distribution in tissues [172]. The common metabolites of catechin are glucuronate conjugates. In enterocytes, catechins have transporter-mediated efflux, which decreases their bioavailability [173]. In the brain, catechins pass through the BBB, but during their transport, they undergo efflux and metabolic transformations due to which their transport efficiency is low [174]. Catechin derivatives have an anti-inflammatory effect in brain cells [175]. In glial cells, catechin and epigallocatechin gallate (EGCG) regulate the kinase events, e.g., by MAPK inhibition, which regulates the TNF-alpha expression. Subsequently, TNFα and NO release is decreased, causing attenuated neuroinflammation [176]. These both also decrease the synthesis of prostaglandin by suppressing COX-2 and downregulate the ROS and RNS [113], thereby giving additional protection to glial cells from ROS and inflammation. Catechins and EGCG can be potential agents for the development of a therapeutic regimen for GBM [177].

### 3.6. Resveratrol

Resveratrol (3,4′,5-trihydroxystilbene) (RES) is found in many plants including grapes, vegetables, peanuts and mulberries. It is a natural polyphenolic phytoalexin. In recent years it has attracted much attention due to its exciting therapeutic effects like antioxidant and anti-inflammatory activities. It penetrates the blood-brain barrier showing neuroprotective effects [179].

In brain cancer, there are mutated forms of isocitrate dehydrogenases (IDHs). In mitochondria, NAD (+)-dependent IDHs play a vital role in the NADH production in the Krebs cycle. Mutated IDH blocks the differentiation of glioma stem cells by forming an increased amount of 2-hydroxyglutaric acid, regulating VEGF, that promotes tumor microenvironment, and also forms the increased HIF-1α leading to glioma invasion. RES maintains IDH levels in stroke and myocardial infarction models. It has also been found that RES activated a mitochondrial complex I and reduced NADH activity in liver cells. In phase I clinical trial, it was found that that administration of RES for 29 days (0.5 to 5 g/day) caused low IGF-1 and IGFBP-3 systemic levels, which demonstrated the antiproliferative role of RES. In the U251 human glioma cell line, RES was also found to activate caspase-3, by inducing apoptosis [180].

Furthermore, in U373MG glioma cells, RES decreased the TNF-α induced invasion, by regulating the activation of NF-𝜅B [181]. RES exerts cell death in GBM cells via apoptosis, autophagy and senescence. Another study on GBM cell lines showed that RES inhibited G2/M arrest followed by senescence induction. Another evidence showed that postoperative administration of resveratrol leads to suppressed tumor growth via apoptosis induction and STAT3 inactivation in advanced orthotopic glioblastoma rats, thus efficiently improving the prognosis. Resveratrol cytotoxic and antiproliferative functions have been associated with inhibition of JAK/STAT pathway. Resveratrol was also found to suppress the function of Src tyrosine kinase, which consequently blocked STAT3 action. In human epidermoid carcinoma (A431) cells, resveratrol obstructed JAK phosphorylation and blocked STAT1 phosphorylation. It regulates the activity of caspase 3 and regulates the apoptotic pathway [192].

Glioma cell lines derived from GBM patients blocked cell proliferation, decreased motility and increased cellular mortality by modulating the Wnt cascade [182]. These observations indicated that RES possesses potent anti-cancer activity and may be used as a potent adjuvant compound in the concomitant regimen for cancer.

### 3.7. Retinoids

Retinoids derive from vitamin A having fat-soluble nature [184,185]. In GBM, they potentially increase the efficacy of chemotherapy and radiotherapy. It is transported in the form of retinol-binding protein from the liver to the target tissues and is then converted to retinaldehyde and concurrently there is a formation of retinoic acid [184,186]. The retinoid pathway plays a vital role in dendritic growth, neurogenesis and cognitive functions [185]. In human glioblastoma multiforme primary cultures, retinoid blocked cell proliferation and migration [185,186]. Retinoids cannot induce apoptosis, however the synthetic analog trans-retinoic acid and N-(4-hydroxyphenyl) retinamide (4-HPR) presented both antiapoptotic and antiproliferative activity [186]. The 4-HPR used with survivin knockdown, an overexpressed pro survival protein in GBM, in around 80% of cells were apoptotic and reduced tumor vascularization, as revealed by in vivo angiogenesis studies. In another study, retinoids induced the differentiation of astrocytes and inhibited telomerase activity [187]. Moreover, retinoid treatment decreases the inflammatory factors, thereby increasing the sensitivity of GBM cells to radiotherapy [185].

The most important characteristics of the phytoconstituents are summarized in Table 1 and the signal transduction pathways targeted by phytoconstituents in glioblastoma are depicted in Figure 3.

## 4. Novel Drug Delivery Approaches

Diverse novel drug delivery approaches are making a significant contribution to cure various types of cancer and many of these systems can be applied to GBM.

### 4.1. Nanoparticles (NPs)

In CNS, GBM is the most encountered of gliomas and there is a pressing need to develop a promising drug delivery system mainly meant to target GBM. The nanoparticles (NPs) approach provides the platform for gene therapy, treatment, diagnosis and monitoring the response. The blood-brain barrier (BBB) is mainly tight junctions present between the brain endothelial cells that mainly control the flow of nutrients, ions, cells towards the brain [194,195]. This restricted drug permeation towards the GBM cells, allows the cancer stem cells to develop therapeutic resistance by evading drug cytotoxicity. Due to increased size, hydrophobicity and efflux by MDR pumps the delivery of chemotherapeutic agents is poor.

Thus, there is a pressing need for targeted and efficacious therapeutic modalities that can cross the BBB and reach the tumor site in the brain [196,197]. A few proposed modalities that can hijack the BBB include passive permeation of lapidated drugs, drug-loaded nanocarriers and prodrugs [198]. NPs having a size below 100 nm, can penetrate even into small vessels while the larger size of NPs develops immunogenicity and encourages their release through the reticular endothelial pathway, thus favoring targeted delivery to cancer cells. NPs formulated with polysorbate (Tween) prompt their transmission across the BBB.

Novel theranostic nanocarriers enable disease treatment but are still complicated [199]. For malignant tumor chemotherapy, there is a development of a multifunctional dendrimer-based theranostic nanosystem focusing on specificity. Doxorubicin, an antitumor drug combined with fifth generation polyamido-amine dendrimer, was associated with acid-sensitive cis-aconityl, and folic acid leading to the formation of the G5 dendrimer. Development of Au NP dendrimers, NHAc-FA-DOX conjugated mixtures entrapment was done with gold nanoparticles. They can be used for chemotherapy and CT imaging of various malignant tumor cells. Temozolomide (TMZ), is first-line chemotherapy treatment for GBM [200]. It is associated with many disadvantages such as decreased bioavailability, increased systemic toxicity compared to other chemotherapeutic agents thereby, TMZ nanoparticles are formulated with poly(lactic-co-glycolic acid) for its delivery. GBM cells overexpress the transferrin receptor, therefore nanoparticles were formulated with a monoclonal antibody for the enhanced antitumor role and enhanced cellular internalization in GBM cells.

Zinc oxide (ZnO) NPs were covalently bound with blood proteins, fibrinogen, albumin and apo-transferrin, then followed by nonspecific adsorption onto ZnO NPs to estimate their consequences for GBM cells. Cell cycle function is disturbed by albumin with c-ZnO NPs and apo-transferrin and thereby decreases the necrotic cell death rate, thus demonstrating the therapeutic outcome on GBM cells. Curcumin is a curcuminoid derivative and a potent anticancer drug through multiple mechanisms that include anti-inflammatory activity, antimitogenic effects, pro-apoptotic effects, immune modulation and has antiangiogenic activity [201]. It influences various factors in brain cancer, such as STAT3, IGF, MAPK, nuclear factor-kappa β (NF-κβ), serine-threonine protein kinase (Akt) and Notch pathways. Curcumin-loaded magnetic NPs can suppress GBM tumor cell proliferation thereby act as a novel drug delivery system and showed improved cytotoxic effects [202].

In the GBM, enzyme histone deacetylases (HDACs) play an important role. Enzyme inhibition can be achieved by chromatin modulation of this enzyme potentially causing apoptosis and cell arrest. Quisinostat (HDACs inhibitor) signifying poor drug delivery thereby NPs of poly(D, L-lactide)-methoxy-poly(ethylene glycol) loaded Quisinostat were formed for orthotopic GBM. Nanomedicine has numerous appealing features that combat GBM such as diffusion through the BBB, targeting GBM tumors and an increased anti-tumor activity via different local mechanisms (immune system stimulation, ROS generation) [202].

However, there are still some barriers to target GBM treatment, due to the lack of preclinical models resembling human GBM, the problems in conducting clinical trials on the human population to reach statistical significance, clinical trials designed to treat advanced-stage GBM and the too late GBM detection. To develop a potent therapeutic regimen against GBM, the aforementioned hurdles need to be overcome [203].

### 4.2. Gliadel Wafers

GBM tumor recurrences are inevitable despite surgical resection following radiotherapy and chemotherapy. Targeted drug delivery is a major challenge due to the presence of BBB and systemic toxicities [204]. Gliadel wafers is a novel approach for the targeted delivery of chemotherapeutic drugs to GBM cells. Approved by FDA for glioblastoma treatment in 1995, Gliadel wafers loaded with carmustine are implanted in the resection cavity, and the drug is released gradually for 2–3 weeks period, as the wafer polymer degradation occurs. Many studies have shown that Gliadel wafers improved drug delivery, reduced systemic adverse effects in recurrent glioma treatment. However, Gliadel wafers are also associated with adverse effects, such as cerebral edema, perioperative seizures, surgical site infection and hydrocephalus that can also lead to death [205]. Hence, its use for the GBM needs to be revaluated.

### 4.3. Drug Delivery in Brain Tissue Using Cellular Carriers

Cell-mediated targeted delivery of chemotherapeutic drugs and nanomaterials represents the modern progress in the drug delivery approach for GBM. Therapeutic drugs are taken up spontaneously by cells or membrane encapsulation occurs, that targets the drug directly to the brain cancer cells [206]. This novel anticancer formulation of cellular cargo having biomimetic nature has a stealth effect on exposure to the systemic circulation [207]. These cell-derived cargos may avoid various adverse reactions compared to synthetic drugs for systemic drug delivery. These susceptible host reactions include premature clearance and protein corona formation.

With numerous advantages, multiple types of cells and components of cellular origin are involved for novel DDS for cancer treatment erythrocytes, leukocytes, platelets and stem cells. In the context of GBM, the following studies had shown potential in GBM models. Postoperative treatment for recurrent malignant glioma was given by cell-mediated anticancer drug delivery approach [208]. The neutrophil inherent ability to traverse BBB paclitaxel-loaded cationic liposomes (PTX-CL) was suspended within isolated neutrophils. Despite the PTX-CL sustained stability, the importance of this delivery approach lies in the anticancer Trojan-horse drug delivery.

In another study, neutrophils loaded coumarin-6 (Cou6)-liposomes effectively penetrate the deep tumor. On the contrary Cou6 alone or the liposome alone when administered remained at the periphery. In in vivo studies, a cell-mediated drug delivery system was proven to be efficacious as tumor recurrence was very slow and there was a significant improvement seen in survival rate [209].

### 4.4. BBB Transient Disruption for Enhanced Drug Delivery

The efficacy of drug delivery systems to the tumoral regions can be improved, by adopting a different strategy to disrupt the integrity of BBB for a time period. Vascular endothelial cells can be stimulated by the external force and loosen junctions that allow the infiltration of chemotherapeutic drugs in the brain cells. Biologic antitumor drugs like antibodies are allowed to couple with stimuli-influenced BBB opening because of their immanent size [210].

Nanomaterials can also be concomitant with the BBB preconditioning for their increased uptake within the tumor tissue [211]. Ultrasound (commonly used clinically) has proven to be effective in BBB disruption transiently. A synergism of BBB preconditioning induced by ultrasound with the delivery of the drug is a potential approach for GBM. The chemotherapeutic efficacy of chemotherapeutic nanoliposomes combined with the ultrasound leads to increased efficacy [212]. The BBB cellular tight junctions can be disrupted reversibly by inducing low-intensity focused ultrasound (LIFU). Intravenous liposomes loaded with TMZ injected to the brain glioma region via disrupted sites of the BBB. LIFU-induced BBB opening was an efficient drug delivery approach, and its confirmation was done in a dual-modal fashion through the high signal intensities of the MR contrast as well as by infrared dye in the target brain region. Mannitol leads to the osmotic opening of the BBB that enhanced the penetration of antibodies across the BBB.

Glioma-bearing mice when administered 25% mannitol transiently open the BBB and lead to the intra-arterial therapeutic antibodies delivery against GBM [213]. For tracking, an anti-GBM antibody, Bevacizumab with deferoxamine containing the PET agent 89Zr was used. When comparison was made with the intraarterial drug delivery with deficient hyperosmolar preconditioning, the proposed approach demonstrated markedly raised PET-tagged antibodies accumulation in the ipsilateral hemisphere region of the brain. Moreover, in intravenous delivery, efficiency was also found to be improved by the BBB hyperosmolar opening. Even though, antibody delivery through the intraarterial and intravenous administration is efficient, further studies are needed. The clinical use of this strategy has some limitations. Firstly, there is a need for specialized equipment (focused ultrasound) that can provide enough external stimulation at the site of GBM [214]. Secondly, the transient opening BBB approach utilizing internal stimuli-responsive materials mainly needs pre-emptive invasive chemical administration. Moreover, adverse effects due to non-specific drug release in the brain regions can also result. Thereby, current studies mainly focus on the safety of the method and upgrading efficacy on delivery [215].

### 4.5. Intra Tumoral Injection of Drug-Loaded Vehicles

In GBM treatment, the intra tumoral drug delivery system is the local administration of therapeutic modalities by injecting the drug directly into the tumor site. This method has acquired scientific attention as increased drug content can be reached at the tumor site with reduced side effects and toxicity, as well, the exposure to normal cells will also be decreased [216]. Even though, the clinical data on this technique is still narrow due to the method invasiveness, method of administration that can lead to severe side effects like bleeding, infections and neuronal damage. Moreover, the diffusion of the drug in GBM target site is very low, since its penetration highly depends on the drug concentration gradient. Thereby, high penetration with no damage and good material biocompatibility is needed for better results [217]. Drugs loaded in injectable hydrogel can directly be injected into the brain through the needle and near the injection site, it is gelled for sustained release.

Recently, many studies have presented the injectable lipid nanocapsules which include the hydrogel incorporated with 4-(N)-lauroyl-Gemcitabine (GemC12). Encapsulating gemcitabine (modified) in lipid nanocapsules leads to the formation of GemC12 [218]. This hydrogel does not have a gelling agent or any external stimuli, these findings clearly show sustained drug release for thirty days in vitro. Further verification of these advantages was done using GBM models in vivo, such as the orthotopic xenograft mouse model and subcutaneous human GBM model [219].

The results demonstrated that promising therapeutic efficiency of GemC12 lipid hydrogel composite within situ gelation property with enhanced therapeutic potential and reduced systemic toxicity was developed and investigated for chemoimmunotherapy. The formulation is composed of the chemotherapeutic drug (for immunogenic death of cancer cells), alginate (polymer for hydrogel) and immune adjuvant (boosting immune response) [219,220].

Following intra tumoral injection, the polymer alginate in the presence of calcium ions forms hydrogel within the tissue site. Hence the encapsulation of chemo and immune drugs can be done physically in the hydrogel network that acts as the pool for sustained drug release. Further, this pool can be used in pharmaceutical techniques by nanocapsules to avoid reoccurrence. In in vivo orthotopic brain tumor model, this cocktail approach demonstrated antitumor immune responses with improved survival rate. Even though the above-mentioned methods represent tremendous advancement in GBM treatment, still many issues remain unclear [218]. In cell-mediated delivery rigorous studies are in demand, to corroborate whether cellular components derived stealth carriers do evade host reactions during circulation. Various unforeseeable variables provoke the disease that includes the type of drug and administration method [209].

There is a need for establishing a preclinical research model, even the amalgamation of clinical therapies should be checked, as no single strategy can counter the high mutation rate and variability of GBM. [209].

###  4.6. Vasculature Targeting via Antibodies

Within the tumor vasculature, antibodies have the ability to selectively target endothelial cells, and thereby these vascular targeting programs have become a foundation in oncology drug development. There are numerous reasons why vascular targeting approaches are interesting such as (i) tumor neovasculature markers are easily accessible to derivatives of i.v.-administered antibody; (ii) neovasculature markers are mainly produced by endothelial cells, and such cells are more stable than malignant cells; (iii) there is rising evidence that damage of the tumor neovasculature causes massive death of tumor cells, which depends on blood vessels for supply of nutrients and oxygen to meet their metabolic needs [221].

In antibody drug conjugates (ADCs), antibody is conjugated to a cytotoxic drug through a linker. Therapeutically, in ADCs an antibody acts as a vehicle to deliver a cytotoxic drug particularly to the tumor tissue site, by targeting an antigen present on the surface of a cancerous cell. ADCs work similar to the prodrugs that need drug release for activation, which mainly occurs after ADC internalization into the target cell. This provides improved tumor-to-normal tissue ratios of ADC drugs when compared to systemic chemotherapy. Many pre-clinical studies have clearly shown that ADCs increase the antitumor activity of “naked” antibodies and decrease the systemic toxicity of the cytotoxic regimen conjugated to the antibody [222,223].

Therapeutic modalities employing antibodies to target angiogenesis can be subdivided into two categories. In the first approach, antibodies are required to deliver therapeutic molecules to the vasculature. In the second, antibodies possess intrinsic antiangiogenic activity, e.g., the inhibition of important vascular proliferation mediators. Prominent examples (recently in clinical trials) are antibodies against a VEGF receptor or αvβ3 and integrin neutralizing anti-VEGF antibodies. When the therapeutic molecules are targeted to tumor blood vessels it results in destruction of these blood vessels and ultimately leads to tumor cell death [221]. Previous work had shown that antibodies are directed against an endothelial cell (artificially induced marker), to deliver a toxin (ricin A) to the tumor neovasculature. Complete tumor remissions were seen in a significant proportion of the mice treated [224].

In a rabbit ocular angiogenesis model, Anti-ED-B antibody fragment scFv (L19) particularly localizes to new blood vessels formed during cancer development. When it is chemically coupled to a photosensitizer and irradiated with red light, this immunoconjugate leads to the complete and selective occlusion of ocular neovasculature and promotes corresponding endothelial cells apoptosis [225].

Tenascins, comprising a family of four extracellular matrix glycoproteins and immunohistochemical analysis in aggressive brain tumors, revealed that the C-terminal domain of tenascin-C is overexpressed with a prominent perivascular staining pattern. In preclinical models of human carcinomas, G11 and F16 human antibodies were shown to interact with tumor vascular specific isoforms leading to anti-tumor effects [226].

For the novel drug linker selection, two different approaches can be considered for vascular targeting purpose. The first strategy is the design of successful drug linker with optimal properties for ADCs and it is based on random screening of chemical libraries. An alternative effort is based on rational drug design principle, taking advantage of the rapidly evolving pathophysiological alteration between normal and tumor vasculature, employing proteomic and genomic strategies. Alternatively, an avenue towards the drug delivery to the vicinity of blood vessels, involved the sub endothelial extracellular matrix (ECM), followed by hydrolytic release of the drug. The recent advancements that determine the gene expression changes within tumor vasculature coordinated with the novel technologies using cross species reactive targeting vehicles and improvements in drug linker technology provide the basis for continuous development of novel ADCs with improved activities in patients with tumors [226]. Current studies have shown that it is possible to selectively target in vivo angiogenesis by means of specific antibodies. The future clinical trials are needed to show its importance for the diagnosis and therapy of angiogenesis in GBM. Delivery systems targeting glioblastoma are presented in Table 2.

## 5. Conclusions

GBM is a deadly malignant tumor having poor prognosis, and novel therapeutic modalities are needed to refine the patient prognosis. There is an abnormal formation and intensive proliferation of vascular structures in GBM causing persistent relapses and resistance to treatments available. ROS can damage DNA or may also cause mutations in its repair events. These components can interact with proteins, lipids and carbohydrates inducing an imbalance in redox homeostasis. Many dysregulating signaling mediators have been found that trigger the ROS levels, and previous studies have shown that various agents induce ROS stimulation in cancer. GBM genomic analysis uncovered various dysregulations in vital cellular events that constitute potential targets for therapeutic development. A targeting strategy that is aimed at multiple components of several signaling pathways, can be advantageous in eradicating GBM.

Natural products are commonly known for their varying molecular and cellular mechanisms at several sites of tumorigenesis. Hence, they would be considered ideal for GBM, either alone or combined with chemotherapeutic agents. This review highlights the anti-glioblastoma potential of natural compounds, and they could be the alternative effective approach for glioblastoma treatment. Clinical trials are still undergoing further evaluation. Even though in the future, preclinical and clinical studies are imperative to elucidate the effects and mechanisms of natural compounds in better targeting resistance and synergistically improving the available chemotherapeutic standard treatments.

Novel drug delivery systems were also discussed, including the role of nanoparticles, Gliadel wafers, drug delivery using cellular carriers, BBB transient disruption for enhanced drug delivery, drug-loaded vehicles direct intra tumoral injection, etc. The main challenge of GBM therapy is the presence of BBB and associated inadequacy of drug delivery. Clinical trials design to treat advanced-stage GBM, and the too late GBM detection is needed. For the GBM treatment the diverse field should be converged (e.g., nano composites soft electronics, and hydrogels). However, before the implementation, a thorough understanding of individual therapy is needed to achieve positive/negative effects. Hence the strategies that will be developed in the future must consider these issues, and more advanced techniques could be developed to improve therapeutic efficacy by understanding GBM deeply.

Fundamental material research, devices and their use should also be harnessed. Despite the major challenges given above, these advancements could lead to a new path for GBM obliteration. The novel therapies could solve the impending problem leading us on the new track for the destruction of GBM.

## Figures and Tables

**Table 1:** Overview of phytoconstituents including structure, target mechanism and limitations.

Compound Structure	Target Mechanism	Limitations	Ref.
Quercetin 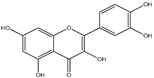	↑caspases 3 and 9 ↓survivin expression, antiapoptotic proteins ↓NFκB-associated PLD1 expression↓HSP27 ↓JAK/STAT3, MMP-2 secretion and cyclin D1 expression Inhibition of Bcl-xl, Bcl-2 and cytochrome c.	Clarification of the mechanism of action as a single therapy or in combination with standard chemotherapeutic therapies is still needed.	[129,130,131,132,133,134,135,136,137,138,139,140,141]
Chrysin 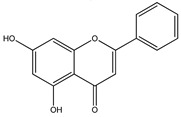	↓ErK/Nrf2 pathway ↓Nrf2, NADPH quinine oxidoreductase-1 and heme oxygenase-1 ↑glutathione peroxidase, superoxide dismutase and catalase activity modulation of MAPK/ERK and P38 induction of caspase-3 and 8.	Chrysin has reduced bioavailability due to its acute metabolism, novel dosage forms, like nanoparticles micelles and liposomes as carriers are needed to increase its bioavailability.	[142,143,144,145,146,147,148,149,150,151]
Luteolin 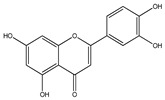	↓iNOS expression ↑miR-7-1-3p, ↓PKCα ↓IL-1β, p65, NF-κB, c-Jun amino-terminal kinase ↓p-AKT ↑caspases 3 and 8.	Clarification of the mechanism of action and clinical research is still needed.	[152,153,154,155,156,157,158,159]
Genistein 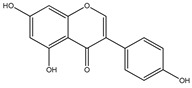	↓tyrosine kinase mediated signaling ↑p53 and p21 ↓cyclin B and cyclin D1 and TERT ↑caspase-3,9.	Clinical trial studies during the different stages of GBM are needed. Mechanism of action is needed to be explored.	[160,161,162,163,164,165,166,167,168,169]
Catechins 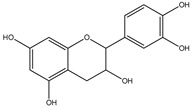	↓MAPK ↓TNFα and NO ↓NF-κB ↑caspase-8.	Studies are needed to define its use in clinical treatment.	[170,171,172,173,174,175,176,177,178]
Resveratrol 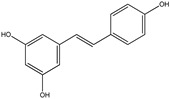	Regulates isocitrate dehydrogenases regulation of STAT3 pathway ↓survivin and antiapoptotic proteins ↓NF-κB signaling ↑caspase 3.	Improvement in solubility, efficient and effective intravenous delivery, first-pass metabolism reduction and enhancement of bioavailability are needed to be studied.	[179,180,181,182,183]
Retinoids 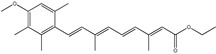	Regulates cyclin D and cyclin D1 proteins, mediates intracellular and extracellular signaling proteins like, ICAM1, cadherin 6, FLRT1 ITGB3.	Details of the molecular mechanism and clinical trial data are needed.	[178,184,185,186,187]

**Table 2:** Drug delivery system targeting glioblastoma.

Delivery Systems	Characteristics	Advantages for the Treatment of GBM	Ref.
Nanoparticles	They are made up of biodegradable polymers conjugated to drugs and antibodies to recognize cancer cells.	Can penetrate even into small vessels, while the larger size of nanoparticles develops immunogenicity and encourages their release through the reticular endothelial pathway (thus favoring targeted delivery to cancer cells).	[194,195,196,197,198,199,200,201,202,203]
Gliadel wafers	A biodegradable polymer of carmustine (1,3-bis [2-chloroethyl]-1-nitrosourea [BCNU]), supplying a controlled release gradually for 2–3 weeks period.	Improved drug delivery, reduced systemic adverse effects in recurrent glioma treatment.	[204,205]
Drug Delivery in Brain Tissue using Cellular Carriers	Cell-mediated targeted delivery through erythrocytes, leukocytes, platelets and stem cells.	Effectively penetrate deeper into the tumor, tumor recurrence was very slow, improved survival rate.	[206,207,208,209]
BBB transient disruption for enhanced drug delivery	Disrupting the integrity of BBB with low-intensity focused ultrasound (LIFU).	Increase the bioavailability and therapeutic efficacy of drugs.	[212,213,214]
Intra tumoral injection of drug-loaded vehicles	They are made up of polymer alginate with calcium ions, forming hydrogel within the tissue site.	Enhanced therapeutic potential and reduced systemic toxicity.	[219,220]
Vascular targeting via antibodies	Antibody is conjugated to a cytotoxic drug through a linker.	Damage of the tumor neovasculature causes massive death of tumor cells.	[221]
