# Correction: Behl et al. Current Perspective on the Natural Compounds and Drug Delivery Techniques in Glioblastoma Multiforme. *Cancers* 2021, *13*, 2765

**DOI:** 10.3390/cancers18030378

**Published:** 2026-01-26

**Authors:** Tapan Behl, Aditi Sharma, Lalit Sharma, Aayush Sehgal, Sukhbir Singh, Neelam Sharma, Gokhan Zengin, Simona Bungau, Mirela Marioara Toma, Daniela Gitea, Elena Emilia Babes, Claudia Teodora Judea Pusta, Adrian Gheorghe Bumbu

**Affiliations:** 1 Department of Pharmacology, Chitkara College of Pharmacy, Chitkara University, Rajpura 140401, India; aayush18004.ccp@chitkara.edu.in (A.S.); sukhbir.singh@chitkara.edu.in (S.S.); neelam.mdu@gmail.com (N.S.); 2 Department of Pharmacology, School of Pharmaceutical Sciences, Shoolini University, Solan 173229, India; aditisharma31790@gmail.com (A.S.); lalitluckysharma88@gmail.com (L.S.); 3 Department of Biology, Faculty of Science, Selcuk University, Konya 42130, Turkey; gokhanzengin@selkuc.edu.tr; 4 Department of Pharmacy, Faculty of Medicine and Pharmacy, University of Oradea, 410028 Oradea, Romania; mmtoma@uoradea.ro (M.M.T.); dgitea@uoradea.ro (D.G.); 5 Doctoral School of Biological and Biomedical Sciences, University of Oradea, 410087 Oradea, Romania; 6 Department of Medical Disciplines, Faculty of Medicine and Pharmacy, University of Oradea, 410073 Oradea, Romania; eebabes@uoradea.ro; 7 Department of Morphological Disciplines, Faculty of Medicine and Pharmacy, University of Oradea, 410073 Oradea, Romania; cjudeapusta@uoradea.ro; 8 Department of Surgical Disciplines, Faculty of Medicine and Pharmacy, University of Oradea, 410073 Oradea, Romania; abumbu@uoradea.ro

## Reference Removal

In the original publication [1], Reference 99 was found to be irrelevant to the content in which it was cited. As recommended by the Editorial Office and Editors-in-Chief, the previously cited Reference 99 has been removed and replaced with an appropriate new reference that accurately supports the content.

The authors confirm that the new added Reference 99 does not affect the scientific conclusions and numbering of the manuscript. This correction was approved by the Academic Editor. The original publication has also been updated.

The new reference is

Budanov, A.V.; Sablina, A.A.; Feinstein, E.; Koonin, E.V.; Chumakov, P.M. Regeneration of peroxiredoxins by p53-regulated sestrins, homologs of bacterial AhpD. *Science* **2004**, *304*, 596–600.

## References

[1] Behl T., Sharma A., Sharma L., Sehgal A., Singh S., Sharma N., Zengin G., Bungau S., Toma M.M., Gitea D. (2021). Current Perspective on the Natural Compounds and Drug Delivery Techniques in Glioblastoma Multiforme. Cancers.

